# Burnout among healthcare providers in the complex environment of the Middle East: a systematic review

**DOI:** 10.1186/s12889-019-7713-1

**Published:** 2019-10-22

**Authors:** Z. Chemali, F. L. Ezzeddine, B. Gelaye, M. L. Dossett, J. Salameh, M. Bizri, B. Dubale, G. Fricchione

**Affiliations:** 1 Departments of Neurology and Psychiatry, Massachusetts General Hospital, Boston, MA USA; 2 Department of Epidemiology, Harvard T.H. Chan School of Public Health, Boston, MA USA; 3 The Chester M. Pierce, MD Division of Global Psychiatry, Massachusetts General Hospital, Boston, MA USA; 4 Benson-Henry Institute for Mind Body Medicine and, Division of General Internal Medicine, Massachusetts General Hospital, Boston, MA USA; 5 Department of Neurology, American University of Beirut Medical Center, Beirut, Lebanon; 6 Department of Psychiatry, American University of Beirut Medical Center, Beirut, Lebanon; 7 Department of Psychiatry, Addis Ababa University, College of Health Sciences, Addis Ababa, Ethiopia; 8 Department of Psychiatry, Benson-Henry Institute for Mind Body Medicine, Massachusetts General Hospital, Boston, MA USA; 9 Harvard Medical School, Boston, MA USA

**Keywords:** Burnout, Middle East, Health personnel

## Abstract

**Background:**

Burnout is a syndrome characterized by emotional exhaustion, increased depersonalization, and a diminished sense of personal accomplishment due to chronic emotional stress at work. Burnout impacts job satisfaction, job performance, vulnerability to illnesses, and interpersonal relationships. There is a gap in the systematic data on the burden of burnout among healthcare professionals from different sectors of healthcare in Middle Eastern countries. Our objective was to examine the burden of burnout among healthcare providers in the Middle East, how it was assessed, which sectors were included, and what interventions have been used.

**Methods:**

Articles were found through a systematic review of search results including PubMed, Web of Science (Thomson Reuters), and PsycINFO (EBSCO) using search terms reflecting burnout in Middle Eastern countries among populations of healthcare providers. Studies were included if they examined a quantitative measure of burnout among healthcare providers in the Middle East.

**Results:**

There were 138 articles that met our inclusion criteria for this systematic review. Studies focused on burnout in the Middle East among physicians (*N* = 54 articles), nurses (*N* = 55), combined populations of healthcare workers (*N* = 22), and medical students (*N* = 7). The Maslach Burnout Inventory was the most common tool to measure burnout. Burnout is common among physicians, nurses, and other healthcare professionals, with prevalence estimates predominantly ranging between 40 and 60%. Burnout among healthcare providers in the Middle East is associated with characteristics of their work environments, exposure to violence and terror, and emotional distress and low social support.

**Conclusions:**

Burnout is highly prevalent among healthcare providers across countries in the Middle East. Previous studies examining burnout in this region have limitations in their methodology. More thoroughly developed epidemiologic studies of burnout are necessary. Health system strengthening is needed in a region that has endured years of ongoing conflict, and there is an urgency to design and implement programs that tackle burnout among health professionals.

## Background

Burnout is a syndrome characterized by emotional exhaustion, depersonalization, and a diminished sense of personal achievement [1]. Evidence suggests that healthcare professionals are especially susceptible to experiencing burnout [2–4], and the rise of burnout prevalence among healthcare providers in recent years has been well documented [5]. Experts in the United States have recently deemed physician burnout as a public health crisis with up to 78% of physicians at least sometimes experiencing feelings of burnout [6]. Studies have also reported high burnout rates among medical students, residents, and nurses, with the prevalence found to be up to 44.2, 45, and 50% respectively [7–9]. Rates of burnout among healthcare workers in other high-income countries have been reported to be the comparable [10–12].

Healthcare providers experiencing burnout may consequently develop symptoms such as anxiety, irritability, mood swings and depression [13–17]. Furthermore, burnout has physical health outcomes including multiple aches and pains, digestive upset, and cardiovascular risks [18–21]. Studies further demonstrate that physicians experiencing burnout are more likely to report job dissatisfaction and intention the leave the medical profession [22]. Lastly, burnout is a concern, as it not only has costly consequences for the provider, but also for the patients and the entire healthcare system. Provider wellbeing is linked to providing quality care and favorable outcomes for patients [23]. Furthermore, the impact of productivity loss related to burnout could lead to fewer healthcare resources that, in turn, can result in healthcare service waitlists and less than optimal healthcare delivery to the public. One estimate of the costs of physician cutback and early retirement suggests it totals to at least $160 million in patient service losses [24].

While there are a number of review studies examining the prevalence and determinants of burnout in healthcare workers in developed and/or westernized countries from North America, Europe, and Australasia [25–30], there is a paucity of reviews focusing on burnout in developing and non-western countries, including Middle Eastern countries. The Middle East may be especially vulnerable to burnout due to the fragmented health systems that are in place. Many Middle Eastern countries have a critical shortage in healthcare professionals often due to brain drain. This may lead to overwork of the available workers, making them prone to burnout. In addition, as an issue specifically pertinent to the Middle East, countries like Iraq, Syria, Palestine and Yemen have been enduring years of ongoing conflict, putting an enormous strain on their healthcare systems as well as their healthcare providers.

### Objectives

This study aimed to systematically review the literature on the burden of burnout among healthcare providers in the Middle East, including the construct of burnout used, which healthcare subspecialties were included, and any interventions evaluated.

This review intends to provide a foundation for future contributions aimed at reducing the burden of burnout in the Middle East.

## Methods

This systematic review was conducted according to Preferred Reporting Items for Systematic.

Review and Meta-Analyses (PRISMA) guidelines (Additional file 1: Table S1) [31].

### Study selection and criteria for inclusion

In PubMed, Web of Science (Thomson Reuters), and PsychINFO (EBSCO), we identified studies using search terms for burnout and Middle Eastern countries (Additional file 2: Table S2). The term “Middle East” has no clear geographic or historical definition due to complex geopolitics [32]. There is controversy on the exact countries that are classified as being in this region, though 16 countries are common in most references, which were included in this study: Bahrain, Egypt, Iran, Iraq, Israel, Jordan, Kuwait, Lebanon, Oman, Palestine, Qatar, Saudi Arabia, Syria, Turkey, United Arab Emirates, and Yemen [33–35]. All articles published prior to March 15, 2019 were eligible for inclusion. Further inclusion and selection criteria has been previously described in a companion article that examines burnout among healthcare providers in Sub-Saharan Africa [36].

### Data extraction and quality assessment

Data extraction and quality assessment methods have been previously described in a companion article [36]. Study quality assessment is presented in Additional file 3: Tables S3, Additional file 4: Table S4, Additional file 5: Table S5 and Additional file 6: Table S6.

## Results

**Fig. 1:**
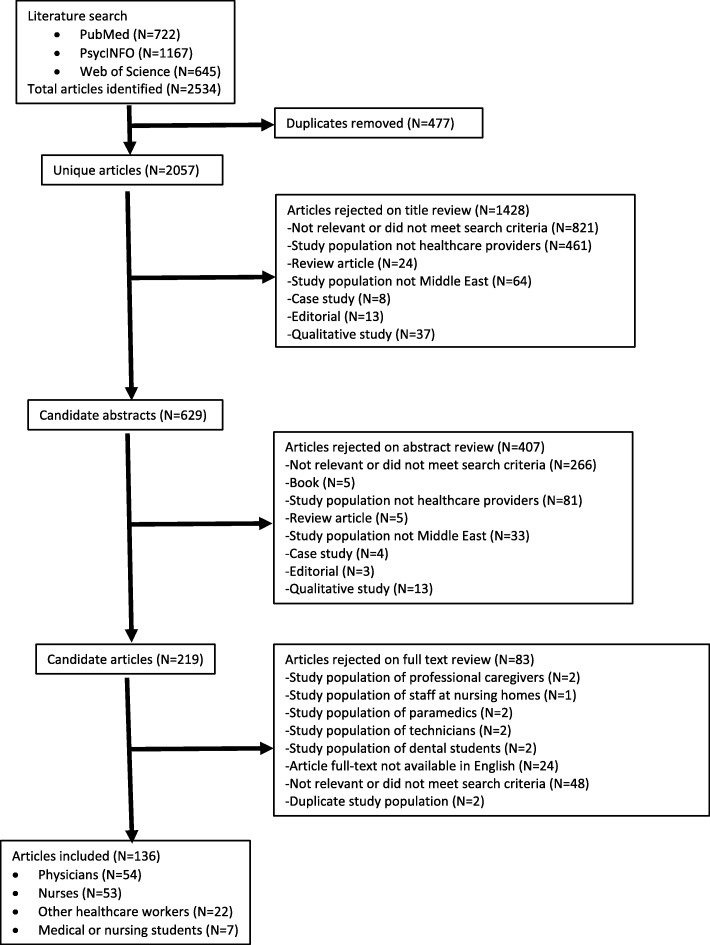
Flowchart of systematic literature review

The original literature search located a total of 722 articles in PubMed, 1167 articles in PsycINFO, and 645 articles in the Web of Science database (Fig. 1). Duplicate articles were removed, and 2057 remained for title review. Articles were rejected on title review for not being relevant or not meeting the search criteria. After reviewing article titles, 629 articles remained for abstract review. Candidate abstracts of the remaining 629 studies were rejected for not being relevant or not meeting the search criteria (*N* = 407). Studies in populations of healthcare providers (*N* = 219) were selected for full-text review. In the full-text review, articles were rejected if they were qualitative studies, not available in English, did not include healthcare providers, or were not relevant to the search criteria (*N* = 83).

A total of 136 articles met our inclusion criteria for this systematic review. Articles examined burnout in the Middle East among physicians (*N* = 54), nurses (*N* = 53), combined populations of healthcare workers (*N* = 22), and medical students (*N* = 7). Six articles examined burnout among healthcare professionals in Egypt, 46 in Turkey, 18 in Saudi Arabia, 29 in Iran, and 19 in Israel, 1 in United Arab Emirates, 3 in Qatar, 1 in Yemen, 8 in Lebanon, 2 in Palestine, 1 in Bahrain, and 2 in Jordan. Of the 138 eligible articles for inclusion, 130 used versions of the Maslach Burnout Inventory to measure burnout. One study in Saudi Arabia examined burnout among National Guard Physicians using the Oldenburg Burnout Inventory. One Israeli study measured burnout among physicians using the burnout subscale of the Professional Quality of Life Scale (ProQOL). Additionally, three Israeli studies measured burnout using the Shirom-Melamed Burnout Questionnaire among physicians (N = 2 studies) and nurses (*N* = 1). Lastly, two studies used the Burnout Measure to measure burnout among medical students in Lebanon.

### Burnout among physicians

Fifty-four articles examined burnout among physicians in Middle Eastern countries, comprising a total of 8588 participants across Egypt, Bahrain, Iran, Israel, Lebanon, Oman, Saudi Arabia, Turkey, Qatar, United Arab Emirates, and Yemen (Table 1). In 49 of the studies, burnout was assessed using the MBI [37–80] or an abbreviated MBI [81–83]. One study used the Oldenburg Burnout Inventory [84], one study used the burnout scale of the ProQOL [85], one study used the Burnout Measure [86], and two studies used the Shirom-Melamed Burnout Questionnaire [87, 88]. One study in Israel used a single-item measure adapted from the full MBI (“How often do you feel burned out from your work?”) to assess burnout on a 7 point Likert scale [89]. Another study in Israel also used a single measure item adapted from the full MBI (“I have become more indifferent towards patients since I started this work”) [90].

**Table 1:** Characteristics of studies on burnout among physicians in the Middle East (*N* = 54)

1st author, Year	Country	Study population	Burnout assessment	EE (%) or mean ± SD	DP (%) or mean ± SD	PA (%) or mean ± SD	Overall burnout (%) or mean ± SD
Abdul-Rahman, 2018 [40]	UAE	Physicians undergoing residency training (*N* = 302)	MBI-HSS	75.5%	84%	74%	84.0%*
Abut, 2012 [41]	Turkey	Trainee anesthesiologists (N = 159)	MBI	Females: 18.1 ± 6.4 Males: 19.2 ± 6.9	Females: 6.1 ± 3.9 Males: 7.4 ± 4.5	Females: 22.8 ± 4.9 Males: 21.1 ± 5.4	–
Afana, 2017 [84]	Qatar	Physicians undergoing residency training (*N* = 142)	AMI	10.7 ± 3.6	5.4 ± 3.9	14.1 ± 3.1	
Agha, 2015 [42]	Saudi Arabia	Doctors working at a tertiary care hospital (*N* = 96)	MBI-HSS	68.8%	63.6%	38.5%	88.5%*
Ahmadpanah, 2015 [43]	Iran	General practitioners at emergency wards (*N* = 100)	MBI	15.4%	14.5%	10.2%	–
Aksoy, 2014 [44]	Turkey	Pediatric residents (*N* = 22) and internal medicine residents (*N* = 33)	MBI	–	–	–	Pediatric residents: 27.3%* Internal medicine residents: 33.3%*
Al-Dubai, 2010 [45]	Yemen	Doctors (N = 356)	MBI	63.2%	19.4%	33.0%	11.7%^
Al-Mendalawi, 2018 [46]	Saudi Arabia	Pediatric residents (*N* = 32)	MBI	43%	72%	41%	–
Al-Sareai, 2013 [47]	Saudi Arabia	Physicians (*N* = 370)	MBI	29.5%	15.7%	19.7%	6.3%^
Al-Shuhail, 2017 [87]	Saudi Arabia	National Guard Physicians (*N* = 95)	The Oldenburg Burnout Inventory	–	–	–	46%^+^
Al-Youbi, 2013 [48]	Saudi Arabia	Pediatricians (N = 130)	MBI	–	–	–	82%*
Aldrees, 2015 [49]	Saudi Arabia	Otolaryngology residents (*N* = 85)	MBI-HSS	29.5 ± 9.6	10.7 ± 6.0	32.33 ± 6.0	–
Aldrees, 2017 [50]	Saudi Arabia	Plastic surgery residents (*N* = 38)	MBI	71%	50%	34%	18%^
Aldrees, 2013 [51]	Saudi Arabia	Physicians (N = 348)	MBI	54%	35%	34%	70%*
Alyamani, 2018 [52]	Saudi Arabia	Medical and surgical residents (*N* = 200)	MBI-HSS	12.5%	31.5%	51.0%	–
Amiri, 2016 [53]	Iran	Primary Care Physicians (*N* = 548)	MBI	15.5 ± 13.6	3.7 ± 5.4	35.5 ± 13.5	17.3%^
Arvandi, 2016 [54]	Iran	Clinical faculty (*N* = 143)	MBI	25%	5.6%	44.8%	–
Ashkar, 2010 [55]	Lebanon	Medical residents (*N* = 155)	MBI-HSS	67.7%	47.1%	23.9%	–
Bar-Sela, 2012 [56]	Israel	Oncology residents (N = 15)	MBI	Pre-intervention: Junior residents:3.6 Senior residents:3.1 Post-intervention: Junior residents: 3.6 Senior residents:1.4	Pre-intervention: Junior residents:2.6 Senior residents:0.98 Post-intervention: Junior residents:2.1 Senior residents:1.4	Pre-intervention: Junior residents:1.3 Senior residents:2.0 Post-intervention: Junior residents:1.9 Senior residents:1.4	–
Bawakid, 2017 [85]	Saudi Arabia	Primary Care Physicians (*N* = 246)	AMI	11.6 ± 4.7	5.6 ± 5.2	14.4 ± 3.6	–
Ben-Itzhak, 2015 [57]	Israel	Emergency physicians (*N* = 70)	MBI	61.4%	51.4%	17.1%	–
Capraz, 2017 [58]	Turkey	Psychiatric trainees (*N* = 167)	MBI	–	–	–	38.3%^+^
Erdur, 2015 [59]	Turkey	Emergency physicians (*N* = 174)	MBI	Males: 24.6 ± 6.0 Females: 24.1 ± 6.7	Males: 30.0 ± 3.4 Females: 29.9 ± 3.9	Males: 11.0 ± 3.2 Females: 10.7 ± 4.1	–
Ghannam, 2019 [86]	Qatar	Medical residents (*N* = 256)	AMI	–	–	–	–
Granek, 2016 [92]	Israel	Oncologists (*N* = 178)	single-item (7-point Likert scale)	–	–	–	Males: 1.84 ± 1.5 Females: 2.69 ± 1.69
Granek, 2017 [93]	Israel & Canada	Oncologists (*N* = 177): Israeli (*N* = 79) and Canadian (*N* = 98)	single-item (7-point Likert scale)	–	–	–	Israeli and Canadian: 1.43 ± 1.6
Grossman, 2019 [60]	Israel	Pediatrics (*N* = 238)	MBI	–	–	–	33%^+^
Gül, 2017 [61]	Turkey	Psychiatrists (*N* = 201)	MBI	Psychiatrists with SCT: 28.9 ± 6.9 Psychiatrists without SCT: 23.7 ± 6.4	Psychiatrists with SCT: 11.1 ± 4.6 Psychiatrists without SCT: 10.4 ± 3.9	Psychiatrists with SCT: 20.3 ± 4.8 Psychiatrists without SCT: 19.2 ± 5.1	Psychiatrists with SCT: 58.4 ± 12.6 Psychiatrists without SCT: 51.4 ± 11.4
Haber, 2013 [88]	Israel	Physicians in conflict zones/situations (*N* = 97)	ProQOL	–	–	–	17.16 ± 5.07
Haik, 2017 [62]	Israel	Burn clinicians (N = 55), specifically clinicians from Burns, Plastic and Reconstruction Surgery and Intensive Care	MBI	24.3 ± 9.3 34.5%	9.3 ± 2.4 27.3%	34.9 ± 3.4 29.0%	38.2%^
Hameed, 2018 [63]	Saudi Arabia	Medical residents (*N* = 181)	MBI	62.2%	70.6%	11.1%	–
Hasan, 2015 [64]	Bahrain	Secondary care doctors (*N* = 230)	MBI-HSS	43.1%	26.7%	51.5%	–
Jalili, 2013 [65]	Iran	Emergency medicine residents and practitioners (*N* = 188)	MBI	22.9	9.3	31.5	–
Jamjoom, 2018 [66]	Saudi Arabia	Pediatric residents (N = 32)	MBI-HSS	43.0%	71.8%	40.6%	–
Karaoglu, 2015 [67]	Turkey	Pediatric residents (*N* = 74)	MBI	23.8 ± 5.3	14.9 ± 2.8	26.7 ± 3.6	–
Keinan, 1987 [68]	Israel	Male internists (N = 79)	single-item (7-point Likert scale)	–	–	–	3.13
Kosan, 2018 [69]	Turkey	Physicians (*N* = 663)	MBI	15.6 ± 7.0	5.7 ± 3.9	21.0 ± 4.4	–
Kotb, 2014 [70]	Egypt	Physicians (*N* = 171): Hospital physicians (*N* = 140) and family physicians (*N* = 31)	MBI	–	–	–	Hospital physicians: 53.9%^+^ Family physicians: 41.9%^+^
Kotb, 2014 [71]	Egypt	Family physicians (N = 31)	MBI	–	–	–	Pre-intervention: 41.9%^+^ Post-intervention 32.3%^+^
Kushnir, 2006 [72]	Israel	Pediatricians (*N* = 126)	Shirom-Melamed Burnout Questionnaire (6-point response scale)	–	–	–	Pediatric specialists: 2.6 ± 0.8 Regular pediatricians 2.6 ± 0.7 Clinic directors: 2.4 ± 0.7 Non-directors: 2.7 ± 0.8
Kushnir, 2008 [91]	Israel	Pediatricians (*N* = 200)	Shirom-Melamed Burnout Questionnaire	–	–	–	2.6 ± 0.8
Kushnir, 2014 [90]	Israel	Primary care physicians (*N* = 136)	MBI	44.5%	36.0%	31.6%	–
Pirincci, 2015 [73]	Turkey	Assistant physicians working at a university hospital(*N* = 222)	MBI	22.3 ± 8.4	8.7 ± 4.7	18.7 ± 5.9	–
Sadat-Ali, 2005 [74]	Saudi Arabia	Orthopedic surgeons (*N* = 69)	MBI	50.7%	59.4%	17.0%	–
Salem, 2018 [75]	Qatar	Primary Care Physicians (*N* = 144)	MBI	11.9%	28.9%	19.7%	16.0%^
Salem, 2018 [76]	Lebanon	Oncologists (*N* = 51)	MBI-HSS	33.3%	19.6%	13.7%	–
Shams, 2013 [77]	Egypt	Anesthesiologists (N = 98)	MBI-HSS	62.2%	56.1%	58.2%	–
Shinan-Altman, 2018 [78]	Israel	Psychosocial oncologists (*N = 85)*	MBI-HSS	16.3%	2.4%	–	–
Soltanifar, 2018 [79]	Iran	Female emergency medicine physicians (*N* = 77)	MBI	84.5%	48.1%	80.5%	–
Talih, 2016 [89]	Lebanon	Medical residents (*N* = 118)	The Burnout Measure	–	–	–	27.0%
Tarcan, 2017 [166]	Turkey	Physicians (*N* = 250)	MBI-HSS	3.6 ± 1.3	2.8 ± 1.2	3.8 ± 1.1	–
Taycan, 2014 [81]	Turkey	Physicians (*N* = 139)	MBI-HSS	14.9 ± 7.0	5.8 ± 3.3	20.4 ± 3.9	
Turgut, 2016 [82]	Turkey	Medical residents (*N* = 127)	MBI	1st year:32.1 ± 6.6 2nd year:32.3 ± 6.1 3rd year:29.6 ± 7.1 4th year:28.1 ± 6.1	1st year: 15.5 ± 3.3 2nd year: 15.9 ± 3.5 3rd year: 14.2 ± 3.1 4th year: 13.9 ± 2.8	1st year: 11.1 ± 6.4 2nd year: 9.5 ± 5.2 3rd year:10.0 ± 5.1 4th year: 8.8 ± 4.6	–
Tzischinsky, 2001 [83]	Israel	Medical residents (*N* = 78)	MBI	–	–	–	Beginning of residency: 2.31 ± 1.3 After 1 year: 2.75 ± 1.37 After 2 years: 2.03 ± 1.27

**Abbreviations**: *AMI* = Abbreviated Maslach Inventory; *MBI* = Maslach Burnout Inventory; *MBI-HSS* = Maslach Burnout Inventory - Human Services Survey; *ProQOL* = Professional Quality of Life which is composed of three discrete subscales. The first subscale measures burnout. *UAE* = United Arab Emirates; *SCT* = Sluggish Cognitive Tempo

*Overall burnout prevalence based on high burnout score in at least one of the burnout categories

^Overall burnout prevalence based on high burnout scores in all three of the burnout categories

^+^Unclear how overall burnout prevalence was defined

High levels of burnout were reported among physicians across the Middle Eastern countries. Among physicians undergoing residency training in the United Arab Emirates (*N* = 302), 70% of the participants reported overall burnout, 75.5% reported moderate-to-high emotional exhaustion, 84% had high depersonalization, and 74% experienced a diminished sense of personal accomplishment [37]. Among doctors working at a tertiary care hospital in Saudi Arabia (*N* = 96), the prevalence of reported high emotional exhaustion, high depersonalization and reduced personal exhaustion were 68.8, 63.6, and 38.5% respectively [39]. A study in Egypt found that more than half of anesthesiologists with academic careers (*N* = 98) met the criteria for all burnout subscales [74]. Among Iranian female emergency medicine physicians (*N* = 85), the level of burnout in the three subscales of emotional exhaustion, depersonalization, and perceived low accomplishment was high in 84.5, 48.1, and 80.5% of participants respectively [76]. A study in Yemen reported that among doctors (*N* = 356), 63.2% were identified as experiencing a high degree of burnout in at least one category of burnout, and those working more than 40 h a week had a 2.8 times higher risk of reporting high levels of burnout compared to those working less than 40 h a week [42]. Among primary care physicians (*N* = 144) in Qatar, the overall prevalence of burnout according to the MBI was 16.1% [72]. A study assessing burnout among Israeli burn specialist clinicians (*N* = 55), 38.2% of them reported high levels of burnout on all three subscales of the MBI [59]. One third of internal medicine residents met the criteria for clinically significant burnout according to an assessment done in Turkey (*N* = 33) [41]. A study conducted in Lebanon found that among physicians undergoing residency training (*N* = 155), 80% of the sample had reported a high level of burnout in at least one domain [52]. Among secondary doctors in Bahrain (*N* = 230), the reported prevalence of high emotional exhaustion, high depersonalization, and diminished sense of personal accomplishment were 43.1, 70.6, and 11.1% respectively [61].

### Burnout among nurses

We found 53 studies that assessed burnout among a total of 10,992 nurses in Palestine, Iran, Turkey, Saudi Arabia, Egypt, Israel, and Jordan (Table 2). All of the 53 studies used the MBI to measure burnout [91–143].

**Table 2:** Characteristics of studies on burnout among nurses in Middle East (*N* = 53)

1st author, Year	Country	Study population	Burnout assessment	EE (%) or mean ± SD	DP (%) or mean ± SD	PA (%) or mean ± SD	Overall Burnout (%) or mean ± SD
Abushaikha, 2009 [94]g	Palestine	Nurses from 5 private hospitals (*N* = 152)	MBI	23.5 ± 10.2	5.4 ± 5.6	34.1 ± 9.4	–
Ahmadi, 2014 [95]	Iran	Nurses from a university affiliated hospital (N = 100): emergency (*N* = 28), orthopedic (N = 22), dialysis wards (*N* = 19), and ICU (*N* = 31)	MBI	–	–	–	Emergency: 17.0%^+^ Orthopedic: 18.6%^+^ Dialysis: 29.0%^+^ ICU: 8.7%^+^
Akkus, 2010 [96]	Turkey	Nurses from stem cell transplantation units (*N* = 57)	MBI	15.1 ± 7.5	3.7 ± 3.3	12.2 ± 5.1	–
Akman, 2016 [97]	Turkey	Pediatric nurses (*N* = 165)	MBI	high job satisfaction scores: 19.1 ± 6.2 low job satisfaction scores: 26.9 ± 6.2	high job satisfaction scores: 5.9 ± 3.9 low job satisfaction scores: 9.2 ± 4.9	high job satisfaction scores: 11.9 ± 5.8 low job satisfaction scores: 9.8 ± 5.1	–
Al-Turki, 2010 [98]	Saudi Arabia	Multi-national nurses from a tertiary care hospital (*N* = 189)	MBI	45.0%	42.0%	29.0%	–
Al-Turki, 2010 [99]	Saudi Arabia	Nurses from a tertiary care hospital (*N* = 37)	MBI	46.0%	49.0%	17.0%	–
Alharbi, 2016 [100]	Saudi Arabia	Critical care nurses (*N* = 150)	MBI	35.2 ± 8.9	16.3 ± 5.2	33.9 ± 8.0	–
Alimoglu, 2005 [101]	Turkey	Nurses (*N* = 141)	MBI	19.2 ± 6.9	5.2 ± 3.6	20.8 ± 4.0	–
Altun, 2002 [102]	Turkey	Nurses (*N* = 160)	MBI	17.3 ± 7.7	4.6 ± 4.3	22.8 ± 5.9	–
Anwar, 2017 [103]	Egypt	Nurses from a university hospital (*N* = 227)	MBI-HSS	24.6 ± 6.8 27%	12.1 ± 4.1 48%	28.4 ± 6.3 78%	–
Arslan, 2016 [104]	Turkey	Nurses from 16 hospitals (*N* = 799)	MBI	16.4 ± 7.1	5.4 ± 4.2	10.1 ± 5.8	–
Azmoon, 2018 [105]	Iran	Nurses from teaching hospitals (*N* = 522)	MBI	13.1 ± 6.6	28.5 ± 9.1	18.4 ± 7.1	–
Bagheri, 2019 [106]	Iran	Nurses from 4 teaching hospitals (*N* = 684)	MBI	38.7%	30.4%	24.6%	–
Bakir, 2010 [107]	Turkey	Military nurses (*N* = 377)	MBI	Age (year) ≤29: 24.8 ± 6.9 Age (year) ≥30: 12.1 ± 10.7	Age (year) ≤29: 10.7 ± 3.7 Age (year) ≥30: 25.3 ± 7.3	Age (year) ≤29: 20.8 ± 5.1 Age (year) ≥30: 20.7 ± 5.5	–
Chayu, 2011 [108]	Israel	Nephrology nurses (*N* = 132)	MBI and Shirom-Melamed Burnout Questionnaire (SMBD)	3.2 ± 1.3	1.8 ± 0.8	2.1 ± 0.9	
Darban, 2016 [109]	Iran	Nurses (*N* = 60)	MBI	–	–	–	Pre-intervention: 60.8 ± 7.8 After: 60.9 ± 7.9 After 1 month: 61.0 ± 8.0 Study group Pre-intervention: 61.1 ± 8.0 After: 58.8 ± 7.6 After 1 month: 54.6 ± 7.0
Demir, 2003 [110]	Turkey	Nurses at university and state hospitals (*N* = 333)	MBI	state hospitals: 19.1 ± 5.7 university hospitals: 17.2 ± 6.0	state hospitals: 5.6 ± 3.6 university hospitals: 4.7 ± 3.1	state hospitals: 20.3 ± 4.1 university hospitals: 20.6 ± 4.1	–
Dor, 2018 [111]	Israel	Hospital nurses (*N* = 278) and community nurses (*N* = 179)	MBI	Hospital: 3.0 ± 1.0 Community: 2.4 ± 0.9	Hospital: 2.2 ± 0.9 Community: 2.0 ± 0.9	Hospital: 2.1 ± 0.6 Community: 2.0 ± 2.7	–
Emold, 2011 [112]	Israel	Nurses from 6oncology units (*N* = 39)	MBI	30.8%	5.1%	15%	–
Farahbod, 2015 [113]	Iran	Nurses at a trauma referral teaching hospital (*N* = 214)	MBI	24.7 ± 12.2	4.7 ± 5.1	41.1 ± 9.6	–
Gholami, 2016 [114]	Iran	Nurses (*N* = 415)	MBI	25.1 ± 12.4	5.9 ± 5.1	33.3 ± 9.6	–
Gunusen, 2010 [115]	Turkey	Nurses (*N* = 108)	MBI	Pre-intervention: Coping group: 20.9 ± 4.7 Control group: 21.4 ± 4.2 Post-intervention: Coping group: 20.9 ± 4.7 Control group: 21.4 ± 4.2	Pre-intervention: Coping group: 5.8 ± 3.6 Control group: 5.0 ± 3.1 Post-intervention: Coping group: 4.6 ± 3.2 Control group: 5.4 ± 2.7	Pre-intervention: Coping group: 19.7 ± 3.4 Control group: 19.7 ± 3.6 Post-intervention: Coping group: 21.4 ± 3.5 Control group: 19.9 ± 3.1	–
Hamaideh, 2011 [116]	Jordan	Mental health nurses (N = 181)	MBI	24.0 ± 13.9 55%	7.0 ± 7.1 50%	31.6 ± 11.5 50%	–
Iecovich, 2017 [117]	Israel	Nurses in long-term care facilities (*N* = 154)	MBI	25.6 ± 10.5	10.2 ± 5.7	20.1 ± 8.1	–
Ilhan, 2008 [118]	Turkey	Nurses (*N* = 418)	MBI	18.0 ± 6.3	5.7 ± 3.9	19.8 ± 4.7	–
Kapucu, 2009 [119]	Turkey	Nurses from hemodialysis units (*N* = 95)	MBI	16.0 ± 6.3	4.7 ± 3.2	21.0 ± 4.6	–
Karadag, 2017 [120]	Turkey	Nurses from a state hospital (*N* = 118)	MBI	48.9 ± 6.1	5.8 ± 3.5	11.2 ± 4.6	–
Karakoc, 2016 [121]	Turkey	Nurses working in dialysis centers (*N* = 171)	MBI	14.0 ± 7.3	4.4 ± 3.4	20.8 ± 4.1	–
Karaman, 2017 [122]	Turkey and Iran	Nurses working in surgical clinics (N = 179) in Turkey (*N* = 87) and Iran (*N* = 97)	MBI	Turkey: 28.3 ± 6.9 Iran: 25.5 ± 9.2	Turkey: 10.2 ± 3.4 Iran: 9.4 ± 3.9	Turkey: 29.5 ± 5.0 Iran: 31.6 ± 5.2	–
Kavurmacı, 2014 [123]	Turkey	Hemodialysis nurses (*N* = 32)	MBI	17.1 ± 8.3	5.9 ± 4.1	20.6 ± 4.1	–
Kutluturkan, 2016 [124]	Turkey	Oncology nurses (*N* = 140)	MBI	(median): 24.0	(median): 9.0	(median): 16.0	–
Kızılcı, 2012 [125]	Turkey	Nurses working in academic institutions (*N* = 94)	MBI	16.4 ± 5.9	4.8 ± 3.6	22.3 ± 4.3	–
Moghaddasi, 2013 [126]	Iran	Nurses working in medical and education centers (*N* = 340)	MBI	22.8 ± 12.4 34.6%	6.99 ± 6.23 28.8%	32.30 ± 9.26 95.7%	–
Mohammad, 2012 [127]	Iran	Nurses (*n* = 712)	MBI	–	–	–	21.9%^+^
Mudallal, 2017 [128]	Jordan	Nurses (*N* = 407)	MBI	31.5 ± 12.8 60.9%	15.2 ± 6.9 65.1%	32.3 ± 18.9 43.0%	–
Naveri, 2009 [129]	Iran	Nurses (N = 200)	MBI	–	–	–	21.7%^+^
Ozbas, 2016 [130]	Turkey	Oncology nurses (*N* = 82)	MBI	Pre-intervention: Study group: 25.3 ± 6.8 Control group: 23.6 ± 6.9 Post-intervention: Study group: 19.0 ± 4.0 Control group: 26.1 ± 5.8	Pre-intervention: Study group: 10.8 ± 4.1 Control group: 9.7 ± 3.0 Post-intervention: Study group: 7.9 ± 2.6 Control group: 11.1 ± 3.1	Pre-intervention: Study group: 29.1 ± 4.6 Control group: 28.4 ± 4.1 Post-intervention: Study group: 30.6 ± 3.7 Control group: 27.7 ± 4.6	–
Ozden, 2013 [131]	Turkey	Intensive care nurses (*N* = 138)	MBI	15.8 ± 7.2	6.5 ± 4.2	20.7 ± 5.0	–
Özgür, 2018 [132]	Turkey	Nurses at a university hospital (*N* = 155)	MBI	20.3 ± 5.9	7.2 ± 3.4	12.76 ± 3.67	–
Palazoglu, 2017 [133]	Turkey	Emergency nurses (*N* = 236)	MBI	21.9 ± 6.5	10.0 ± 4.0	21.0 ± 4.5	–
Rezaei, 2018 [134]	Iran	Nurses (*N* = 200)	MBI-HSS	2.9 ± 0.3	2.9 ± 0.2	2.6 ± 0.5	–
Ron, 2014 [135]	Israel	Nurses (N = 214)	single-item	–	–	–	3.92 ± 0.44
Sabanciogullari 2015 [136]	Turkey	Nurses working at a university hospital (*N* = 63)	MBI	14.8 ± 5.1	24.6 ± 3.7	21.6 ± 2.4	–
Sahraian, 2008 [137]	Iran	Nurses working at public hospitals (*N* = 180)	MBI	25.8 ± 0.9	5.9 ± 0.3	29.6 ± 1.1	–
Shahriari, 2014 [138]	Iran	Critical care nurses (*N* = 170)	MBI	Fixed shift schedule: 60%, Non-fixed schedule: 12.9%	Fixed shift schedule: 32.9%, Non-fixed schedule: 18.8%	Fixed shift schedule: 27.1% Non-fixed schedule: 43.5%	–
Shamali, 2015 [139]	Iran	Nurses with rotating shift schedules (*N* = 130) and fixed shift schedules (N = 130)	MBI	Rotating schedule 20.5 ± 11.1 Fixed schedule: 26.6 ± 11.4	Rotating schedule 10.8 ± 6.7 Fixed schedule: 11.7 ± 6.4	Rotating schedule 31.1 ± 9.9 Fixed schedule: 29.3 ± 11.2	–
Sorour, 2012 [140]	Egypt	Emergency nurses (*N* = 58)	MBI	–	–	–	37.9%*
Soroush, 2016 [141]	Iran	Nurses working in neonatal intensive care units (*N* = 86)	MBI	21.3 ± 8.1	2.6 ± 31	22.6 ± 5.4	–
Taleghani, 2017 [142]	Iran	Oncology nurses (*N* = 67)	MBI	38.1 ± 22.7	25.6 ± 17.8	47.9 ± 13.7	–
Tekindal, 2012 [143]	Turkeys	Nurses working at a state hospital (*N* = 225)	MBI	27.2 ± 6.3	9.3 ± 3.1	29.4 ± 4.2	–
Topbas, 2019 [144]	Turkey	Hemodialysis nurses (N = 82)	MBI	15.1 ± 7.9	8.2 ± 5.1	22.3 ± 4.9	
Tuna, 2014 [145]	Turkey	Oncology nurses (N = 189)	MBI	18.3 ± 6.2	5.9 ± 3.6	11.1 ± 4.1	–
Yousefy, 2006 [146]	Iran	Psychiatric nurses (*N* = 55) and medical nurses (*N* = 51)	MBI	16.64 ± 7.54	4.96 ± 5.50	13.82 ± 9.83	–

**Abbreviations**: *MBI* = Maslach Burnout Inventory; *MBI-HSS* = Maslach Burnout Inventory - Human Services Survey

*Overall burnout prevalence based on high burnout score in at least one of the burnout categories

^Overall burnout prevalence based on high burnout scores in all three of the burnout categories

^+^Unclear how overall burnout prevalence was defined

Nurses reported high levels of burnout (Table 2). For example, among nurses from five private hospitals in Palestine (*N* = 152), burnout measures were high on the emotional exhaustion (mean ± standard deviation (SD): 23.48 ± 10.23), depersonalization (5.42 ± 5.61), and personal achievement (34.14 ± 9.44) subscales of the MBI [91]. A study conducted among Iranian nurses working at public hospitals (*N* = 180) found that 25% of the participants met the criteria for burnout according to the MBI [134]. A study of nurses in Israel (*N* = 39) reported that 30.8% of participants reported emotional exhaustion very frequently, 5.1% have a cynical feeling towards their job often, and 15.4% rarely experience professional self-actualization [109]. Among mental health nurses in Jordan (*N* = 181), 55% of them reported high level of burnout in the subscale of emotional exhaustion, 50% reported high depersonalization, and 50% reported a reduced sense of personal accomplishment [113].

### Burnout among combined populations of healthcare workers

There were 22 articles that studied burnout among combined populations of healthcare workers (Table 3). Ten studies were based in Turkey, six studies in Iran, three studies in Lebanon, one each in Egypt, Israel, and Palestine. All of the 22 studies used the MBI to assess burnout [144–165]. High levels of burnout were reported in these populations. For example, among employees working at an emergency department in Lebanon (*N* = 256), the prevalence of reported high emotional exhaustion, depersonalization, and reduced sense of personal accomplishment was 54.9, 43.5, and 44.5% respectively [148]. A study in Iran on mental health service providers (*N* = 100), burnout scores were high in emotional exhaustion ((mean ± SD): 24.9 ± 6.9), depersonalization (9.3 ± 2.1), and diminished sense of personal accomplishment (35.5 ± 7.0) [150]. Among oncology employees in Turkey (*N* = 90), reported prevalence of emotional exhaustion, depersonalization, and reduced sense of personal achievement was 42, 20, and 35.6% respectively [153].

**Table 3:** Characteristics of studies on burnout among combined populations of healthcare workers in the Middle East (N = 22)

1st author, Year	Country	Study population	Burnout assessment	EE (%) or mean ± SD	DP (%) or mean ± SD	PA (%) or mean ± SD	Overall Burnout or mean ± SD
Abarghouei, 2016 [147]	Iran	Hospital personnel: (N = 340): health sector (N = 170) and administrative sector (N = 170)	MBI	21.7 ± 7.3	9.6 ± 3.7	26.8 ± 6.2	–
Abdo, 2016 [148]	Egypt	Physicians (*N* = 266) and nurses (*N* = 284) at an emergency hospital	MBI	Nurses: 52.8% Physicians: 29.7%	Nurses: 44.4% Physicians: 45.6%	Nurses: 96.5% Physicians: 99.2%	–
Alacacioglu, 2009 [149]	Turkey	Physicians (*N* = 77) and nurses (*N* = 56) at an oncology clinic	MBI	Nurses: 5.4% Physicians: 7.8%	Nurses: 5.4% Physicians: 15.6%	Nurses: 100% Physicians: 100%	–
Alameddine, 2011 [150]	Lebanon	Employees working at an emergency department (*N* = 256):	MBI	54.9%	43.5%	44.5%	–
Alameddine, 2012 [151]	Lebanon	Health providers (*N* = 755)	MBI	23.3%	12.8%	18.7%	–
Alameddine, 2017 [152]	Lebanon	Health providers (*N* = 1000)	MBI-HSS	22.1%	10.8%	42.1%	–
Ashtari, 2009 [153]	Iran	Mental health service providers (N = 100)	MBI	29.4 ± 6.9	9.3 ± 2.1	35.5 ± 7.0	–
Bijari, 2016 [154]	Iran	Rural health workers (*N* = 423)	MBI	17.7%	6.4%	53%	5.7%^
Calgan, 2011 [155]	Turkey	Community pharmacists (*N* = 251)	MBI	16.8	4.0	22.0	–
Demirci, 2010 [156]	Turkey	Oncology employees (*N* = 90)	MBI	23.80 ± 10.98 42%	5.21 ± 4.99 20%	36.23 ± 8.05 35.6%	–
Devebakan, 2018 [157]	Turkey	Healthcare workers (*N* = 80)	MBI	–	–	–	–
Gokcen, 2013 [158]	Turkey	Emergency department workers (*N* = 270): doctors(*N* = 630), nurses(*N* = 85), paramedics (*N* = 122)	MBI	14.3 ± 8.2	6.5 ± 4.3	18.9 ± 6.5.	
Gulalp, 2008 [159]	Israel	Emergency physicians (N = 8) nurses (N = 40), and nurse’s aide (N = 12)	MBI	19.1 ± 9.1	7.8 ± 4.7	22.3 ± 5.9	–
Guveli, 2015 [160]	Turkey	Oncology health workers (*N* = 159)	MBI	14.2 ± 7.2	4.9 ± 3.4	6.2 ± 5.3	–
Hamdan, 2017 [161]	Palestine	Emergency workers: physicians (*N* = 142), nurses (*N* = 161), and administrative personnel (*N* = 141).	MBI-HSS	64.8%	38.1%	34.6%	–
Hosseiniarzfuni, 2015 [162]	Iran	Nurses (*N* = 35) and technicians (N = 32)	MBI	–	–	–	Technicians: 19%^ Nurses: 24%^
Kabir, 2016 [163]	Iran	Healthcare workers (*N* = 1141)	MBI	–	–	–	9.1%^
Kömür 2017 [164]	Turkey	Mortuary staff (N = 142): forensic medicine specialists (N = 40), forensic medicine residents (*N* = 54), autopsy technicians (*N* = 45) and other staff members (*N* = 24)	MBI	11.1 ± 6.6 14%	8.2 ± 3.6 32.4%	17.4 ± 5.5 76.1%	–
Malakouti, 2011 [165]	Iran	Rural mental health workers (Behvarzes) (*N* = 212)	MBI	14.5 ± 9.9	2.2 ± 3.4	33.8 ± 10.4	–
Tarcan, 2017 [166]	Turkey	Health professionals (*N* = 250): doctors (*N* = 38), nurses (N = 55), technicians (N = 118), and medical secretaries (*N* = 39).	MBI-HSS	3.6 ± 1.3	2.8 ± 1.2	3.8 ± 1.1	–
Tekin, 2017 [167]	Turkey	Health care professionals (*N* = 120): nurses (*N* = 73) and doctors (*N* = 47)	MBI	Type D personality (negative): 20.7 ± 7.6 (positive): 16.3 ± 8.7	Type D personality (negative): 6.8 ± 4.2 (positive): 4.5 ± 4.2	Type D personality (negative): 11.7 ± 5.2 (positive): 9.7 ± 5.9	–
Tunc, 2009 [168]	Turkey	Health care professionals (250): physicians (N = 170) and nurses (*N* = 81)	MBI-HSS	Physicians: 1.6 ± 0.9 Nurses: 2.2 ± 0.9	Physicians: 0.9 ± 0.7 Nurses: 1.3 ± 0.8	Physicians: 1.0 ± 0.7 Nurses: 1.4 ± 0.8	–

**Abbreviations:** *MBI* = Maslach Burnout Inventory; *MBI-HSS* = Maslach Burnout Inventory - Human Services Survey

*Overall burnout prevalence based on high burnout score in at least one of the burnout categories

^Overall burnout prevalence based on high burnout scores in all three of the burnout categories

^+^Unclear how overall burnout prevalence was defined

### Burnout among medical students

A total of seven articles examined burnout among medical students in Oman, Saudi Arabia, Iran, Turkey, and Lebanon (Table 4). Among medical students in Oman (*N* = 662), the prevalence of overall severe burnout was 7.4% as assessed by the MBI [166]. Two studies in Saudi Arabia used the MBI and reported that among 249 and 276 medical students, the overall burnout prevalence was 67.1 and 56.5% respectively [167, 168]. A study in Iran found that among 230 medical students, burnout scores were high for emotional exhaustion (mean ± SD: 20.2 ± 10.1), depersonalization (6.6 ± 5.3), and personal accomplishment (34.9 ± 8.6, 169). Among 165 medical students in Lebanon, 75% were reported to exhibit high levels of overall burnout [170]. Another study in Lebanon found that 43% of students (*N* = 176) were experiencing high levels of overall burnout according to the Burnout Measure [171]. Finally, among 302 medical students in Turkey, high burnout scores were reported for emotional exhaustion (25.5 ± 7.5), depersonalization (11.3 ± 3.9), and personal accomplishment (24.7 ± 3.4) [172].

**Table 4:** Characteristics of studies on burnout among medical students in the Middle East (*N* = 7)

First Author, Year	Country	Study population	Burnout assessment	EE (%) or mean ± SD	DP (%) or mean ± SD	PA (%) or mean ± SD	Overall Burnout or mean ± SD
Al-Alawi, 2017 [169]	Oman	Medical students (*N* = 662)	MBI	30.1%	33.9%	31.1%	7.4%^
Almalki, 2017 [170]	Saudi Arabia	Medical students (*N* = 249)	MBI-HSS	62.2%	58.6%	60.2%	67.1%*
Altannir, 2019 [171]	Saudi Arabia	Medical students (N = 276)	MBI	18.5 ± 10.3 17.4%	14.2 ± 9.2 56.9%	28.7 ± 9.5 14.9%	13.4^
Ebrahimi, 2018 [172]	Iran	Medical students (N = 230)	MBI	20.2 ± 10.2	6.7 ± 5.3	34.9 ± 8.6	–
Fares, 2016 [173]	Lebanon	Medical students (*N* = 165)	MBI-HSS	–	–	–	75%^+^
Sevencan, 2010 [175]	Turkey	Medical students (*N* = 302)	MBI	25.5 ± 7.5	11.3 ± 3.9	24.7 ± 3.4	–
Talih, 2018 [174]	Lebanon	Medical students (*N* = 176)	Burnout Measure	–	–	–	43%

**Abbreviations:** *MBI* = Maslach Burnout Inventory; *MBI-HSS* = Maslach Burnout Inventory - Human Services Survey

*Overall burnout prevalence based on high burnout score in at least one of the burnout categories

^Overall burnout prevalence based on high burnout scores in all three of the burnout categories

^+^Unclear how overall burnout prevalence was defined

### Risk factors associated with burnout among healthcare providers

Components in the work environment were statistically significantly associated with measures of burnout, including heavy workload, unorganized and difficult working conditions, difficulties balancing professional and private life, and income. For example, among medical residents in Lebanon (*N* = 155), those who reported working more than 80 h a week were 2.91 times as likely to report burn out compared to residents working less than 80 h a week [52]. In a Saudi Arabia study conducted among primary care physicians (*N* = 246), multivariate regression analysis showed that the number of patients per day (*p* < 0.001), more paperwork (p < 0.001), unorganized patients flow to clinics (*p* = 0.021), and patient pressure/violence (p < 0.001) were all statistically significant positive predictors of burnout [82]. Work overload and difficulties balancing professional and private life were both reported to be significant predictors of high emotional exhaustion (*p* < 0.05) in a study done in Iran [62]. A study in Turkey found among assistant physicians working at a university hospital (*N* = 222), those who considered their monthly income level as “poor” differed significantly from those who regarded their monthly income levels as “good” in terms of mean scores in subscales of the MBI, emotional exhaustion, depersonalization, and personal accomplishment (p < 0.05) [70]. A study in Egypt among 227 nurses in a university hospital reported that the number of shifts and shift timing (night) proved to be significant predictors for high grades of the three domains of burnout collectively among nurses [100]. Among 333 nurses at university and state hospitals in Turkey, always working night was found the be positively associated with all subscales of burnout as reported by the MBI (p < 0.05) as compared to always working day shift or occasionally working night shift [107]. A study in Iran of critical care nurses (*N* = 170) reported that among nurses with fixed shift schedules, the odds of high risk of emotional exhaustion as measured by the MBI was 10.1 (95%CI: 4.68–21.75) times higher than the odds among nurses with non-fixed shift schedule [135].

Exposure to violence, terror, and conflict were all associated with increased odds of burnout. This suggests there may be an interaction between burnout and secondary traumatic stress in Middle East caregivers. The war trauma primary and secondary exposure as being worsening factors in burnout was specifically mentioned and reported in a study related to Lebanese nurses working in academic centers. The study also linked trauma exposure to higher level of depression in nurses (36.2%) compared to 10% of general population [171]. Moreover, among emergency physicians working in Turkey (*N* = 174), there were significant associations between emotional exhaustion, as reported by the MBI, and overall violence (*p* = 0.012) and verbal violence (*p* = 0.016) and depersonalization and total violence (*p* = 0.021) and verbal violence (p = 0.012) [56]. A study done on healthcare workers in Palestine (*N* = 444) reported that the odds of high risk of burnout among those who had exposure to violence was two times the odds among those who had no exposure to violence [158]. Additionally, a study in Israel which assessed burnout among physicians in conflict zones (*N* = 97) reported that higher levels of PTSD symptoms due to war and terror were associated with higher levels of compassion fatigue (β = 0.594; t = 4.419; *p* < 0.001) [85]. Another study in Israel among nurses (*N* = 214) reported that nurses’ exposure to national terror and the level of distress they experienced due to ongoing terror attacks were statistically significantly associated with high levels of burnout [132].

Reports examining the association between age, time in career, and burnout were variable. Overall, results suggest that the association between age and burnout is bimodal, in which younger age (i.e. under 25) and old age (i.e. over 55), along with greater work experience are associated with the highest burnout levels. Many studies suggested that younger age was a significant predictor of burnout. For example, a study among physicians in Saudi Arabia (*N* = 270) found that younger age was significantly associated with higher emotional exhaustion and lower personal accomplishment scores [44]. A study in Turkey found that among physicians (*N* = 663), mean emotional exhaustion and depersonalization scores were significantly higher and personal accomplishment scores were significantly lower in participants aged under 25 years [66]. Among two different studies of nurses in Turkey (*N* = 141) and (*N* = 171) younger age was also found to be associated with increased risk of burnout [98, 118]. Among burn clinicians in Israel (*N* = 55), older age was found to be a protective factor for high risk of overall burnout (OR = 0.79, *p* = 0.019) [59]. Among oncology employees (*N* = 90) working in Turkey, being of age less than 35 years was significantly associated with burnout [153]; however, this study also reported that work experience greater than 10 years was also significantly associated with burnout. In that same vein, other studies reported that increasing age and greater occupational experience was positively associated with burnout. Among psychiatric trainees (*N* = 167) in Turkey, logistic regression confirmed that older age (*p* = 0.02) was associated with severe burnout [55]. Among nurses in Iran (*N* = 200), greater age and work experience accounted for 30% of the variance in depersonalization [131]. A study in Egypt among 266 physicians and 284 nurses also found that age and years of experience were significant predictors of burnout [145]. In Iran among 1141 healthcare workers, the number of years of experience in the health professional was also associated with increased levels of burnout (*p* < 0.001) [160].

The majority of studies that found a significant association between gender and burnout reported that female gender was a significant predictor of increased risk for burnout [38, 48, 52, 81, 89, 103, 105, 117, 125, 163, 165, 168, 173].. For example, among trainee anesthesiologists in Turkey (*N* = 159) and physicians in Saudi Arabia (*N* = 348), female gender was significantly associated with greater burnout scores (*p* < 0.05, 0.02 respectively) [38, 48]. Among nephrology nurses in Israel (*N* = 132) and nurses in Jordan (*N* = 407), female gender was also found to be a significant predictor of burnout (p < 0.05, *p* < 0.01) [105, 125]. Among medical students in Saudi Arabia (*N* = 276), female gender was a significant predictor of emotional exhaustion and depersonalization (OR = 4.34, *p* = 0.001) [168]. Only three studies reported that males were associated with higher burnout risk compared to females: among 130 pediatricians in Saudi Arabia, the prevalence of burnout among males was significantly greater than females (40% vs 31%; *p* = 0.012, 45). Another study in Turkey among nurses working in dialysis centers (*N* = 171) found that male sex was associated with higher depersonalization scores (*p* < 0.05) [118]. Another study in Iran found that among 340 hospital personnel, emotional exhaustion and depersonalization scores were higher among males compared to females [144]. Lastly, some studies reported no association at all between gender and burnout, such as a study among 302 medical students in Turkey [172] and a study among primary care physicians in Iran (*N* = 548) [50].

Experiences of stress and lack of job support were additionally found to be predictive of job burnout among healthcare professionals. For example, among trainee anesthesiologists in Turkey, perceived stress, as measured by the Perceived Stress Scale, were significantly associated with increased odds of burnout (*p* < 0.05) [38]. Among anesthesiologists in Egypt, there was a significant positive correlation between job stress, as measured by Workplace Stress Scale of the American Institute of Stress, and MBI-HSS subscales [74]. Moreover a significant positive relationship between sub dimensions of job stress level, as measured by the Job Stressors Scale, and of burnout level (p < 0.05) was found among oncology nurses in Turkey [142]. Furthermore, the strongest significant single predictor of all burnout dimensions among anesthesiologists in Egypt (*N* = 98) was lack of job support [74]. Among nurses from four teaching hospitals in Iran (*N* = 684), lack of workplace support was also found to be a significant predictor of burnout (β = − 0.043, CI 95% = − 0.097–-0.003) [103].

### Burnout intervention programs

There were only a few programs aimed at alleviating burnout in the Middle East. Only three studies examined burnout-related interventions in physicians [53, 68, 83], and only four examined interventions for nurses [106, 112, 127, 133]. Bar-Sela et al. examined the effect of “Balint” group meetings as an intervention aimed at improving burnout levels among oncology junior and senior residents in Israel [53]. The oncology residents attended nine sessions in one year, in which they meet to discuss cases from the residents’ experiences. From a comparison of MBI scores, the investigators found that higher measures of emotional exhaustion and depersonalization were noted in junior residents at the beginning of the year, before the intervention; however, in senior residents, all measures of burnout increased after the intervention. In Qatar, Ghannam et al. assessed the effect of a stress management intervention on residents’ (*N* = 256) burnout outcomes [83]. They conducted a 1-day workshop with the objectives being to help residents identify stressors, identify early warning signs of stress, and practice intervention techniques. At 1-month post-course, 83.6% listed at least one piece of knowledge or skill that they had put into practice since the course. Using the abbreviated MBI, residents improved in three of the four burnout constructs: emotional exhaustion, depersonalization, and satisfaction with the practice of medicine. Kotb, Mohamed, Kamel, Ismail and Abdulmajeed evaluated the effect of an educational program on level of professional burnout among family physicians working in family practice centers in Egypt (*N* = 31) [68]. Prevalence of burnout after six months of the intervention program decreased from (41.9%) to (32.3%).

In Iran, Darban, Balouchi, Narouipour, Sarfazaei and Shahdahi examined the effect of a communication skills training on the burnout of nurses [106]. The training was carried out for the intervention group as a 2-day workshop for 8 h within a week. They found that the mean score of job burnout in the intervention group significantly decreased while mean scores of burnout in the control group showed no significant change (*p* = 0.01). A randomized controlled trial was conducted in Turkey by Gunusen and Ustun to evaluate the effects of coping and support group interventions on the reduction of burnout among nurses [112]. Cognitive coping strategies and problem-solving methods were used for coping training. The objectives of the social support groups were to provide support, information and a sense of belonging, and create an environment where individuals share their experiences. Right after the intervention, there was an immediate reduction in emotional exhaustion dimension in the intervention group, as assessed by the MBI, of burnout. However, after 6 months, scores were increased again.

Another study in Turkey was conducted to determine the effect of a “psychodrama-based psychological empowerment program” on the levels of burnout in oncology nurses (*N* = 82) [127]. They found that there was a statistically significant difference between nurses’ scores in the sub dimensions of emotional burnout, depersonalization, and personal accomplishment (*p* < 0.05), and such that nurses in the study group had lower levels of emotional burnout and desensitization and high personal achievement scores at one and three months after the psychological empowerment program compared to nurses in the control/comparison group. Lastly, Sabanciogullari and Dogan evaluated the effects of the Professional Identity Development Program on burnout levels of registered nurses in Turkey [133]. The program consisted of ten sessions delivered to the study group once a week. During the research period, burnout levels significantly decreased in the study group, while those of the control group increased.

## Discussion

Burnout is prevalent among physicians, nurses, and other medical professionals in the Middle East. Prevalence estimates range between 40 and 60% with reported rates between 13 and 100% (Tables S1–4). The minority of studies that reported lower than average levels of burnout attributed such differences to the quality of their study, sample size, and characteristics of the population studied. Nurses reported the highest levels of burnout among healthcare providers. High levels of burnout were associated with harsh work conditions, stress, and exposure to violence and conflict. Studies in the Middle East maintain previous research among healthcare providers that have demonstrated that risk factors for burnout include female gender, young age, and low support and resources to handle workload. The number of reported interventions aimed at alleviating burnout in the Middle East is scarce. Nevertheless, the interventions that have been attempted are similar to some that have shown benefits in North America and Europe (e.g cognitive skills (178;179), support groups (180–182), Balint groups (183; 184)). Mindfulness interventions were mentioned anecdotally in several papers that did not meet inclusion criteria for this review. The duration of these interventions varied from a week [109] to a year [56] depending on the format. Repetitive and regular interventions that raise awareness and inform professionals about burnout, emotional empowerment, and stress management may have the potential to render a long-lasting protective effect on the risk of occupational burnout in the Middle East. Therefore, it would be reasonable to encourage studies of contextualized caregiver interventions that address culturally relevant ways to reduce the toxic stress of working with traumatized populations in under-resourced settings while also enhancing resilience factors.

### Limitations of current studies

We noted that the MBI was used as the assessment tool for burnout in the majority of studies. Among the studies that used the MBI, we point out that the burnout scores were presorted in multiple manners either listing the percentage of participants with high burnout on each subscale, or the percentage of participants with high burnout on each subscale and the total score, and the percentage of participants with high total burnout. Some listed the percentage of participants with high burnout on emotional subscale only or the total and individual burnout as continuous scores together or separately. Finally, some studies reported individual burnout as continuous score while others reported individual burnout as continuous scores and percentages of participants. In addition, there were three different versions of the MBI used namely: MBI, MBI-HSS, and Abbreviated MBI (or AMI) rendering difficult the direct comparison of burnout rates in different countries and populations of healthcare workers. Additionally, recent work has raised concerns about how the MBI operationalizes burnout [174]. Having said that, the MBI has not been validated in healthcare workers in the Middle East, and there may be different cultural interpretations of questions related to the construct of burnout.

In addition, Table 3 points to a total of 22 studies examining burnout in combined populations of healthcare workers in the Middle East knowing that those workers have highly variable job workload and responsibilities and should not have been lumped together for precision sake. Last but not least, the cross-sectional studies lacked temporality and varied in quality adding to the limitations of the small sample sizes. (Additional file 3: Table S3, Additional file 4: Table S4, Additional file 5: Table S5 and Additional file 6: Table S6).

## Conclusion

In contrast to high-income countries, the Middle East has not given much attention to burnout, despite the many stressors facing its healthcare community. Given the adverse health effects of burnout on patients and providers alike, the ever-increasing burden of caring for major public health threats, amidst ongoing regional conflicts and refugee crises with a paucity of resources and shortage of support, more attention needs to be paid to healthcare provider well-being in the Middle East. Further studies should include longitudinal assessments tackling cognition, performance and quality of life as well as reports on mood, substance use, and safety issues such as suicide, to enhance our understanding of the burnout syndrome and its consequences in the Middle East. Moreover, efforts should be channeled to assessing burnout consistently with a validated instrument or developing new instruments that would perform culturally better in the context of the Middle East. Finally, designing and deploying programs aimed at raising awareness, promoting well-being and burnout prevention, and improving coping with burnout symptoms through evidence-based stress management and resiliency training in the Middle East would be the gold standard for any academic, private or public institution promoting excellence in care for the population they serve.

## Supplementary information

**Additional file 1: Table S1.** Preferred Reporting Items for Systematic Review and Meta-Analyses (PRISMA) guidelines.

**Additional file 2: Table S2.** Database terms of search.

**Additional file 3: Table S3.** Quality assessment based on modified Newcastle-Ottawa Scale of studies on burnout among physicians in the Middle East (*N* = 54).

**Additional file 4: Table S4**. Quality assessment based on modified Newcastle-Ottawa Scale of studies on burnout among nurses in the Middle East (*N* = 53).

**Additional file 5: Table S5.** Quality assessment based on the Newcastle-Ottawa Scale on burnout among healthcare workers in the Middle East (*N* = 22).

**Additional file 6: Table S6.** Quality assessment based on the Newcastle-Ottawa Scale on burnout among midwives, medical and nursing students in the Middle East(*N* = 7).

## Data Availability

Not applicable.

## Abbreviations

AMI: Abbreviated Maslach Inventory
MBI: Maslach Burnout Inventory
MBI-HSS: Maslach Burnout Inventory - Human Services Survey
PRISMA: Preferred Reporitng Items for Systematic Review and Meta-Analyses
ProQOL: Professional Quality of Life
SCT: Sluggish Cognitive Tempo
UAE: United Arab Emirates
