# Supplementary material for: Burnout among healthcare providers in the complex environment of the Middle East: a systematic review
Source: BMC Public Health. 2019 Oct 22;19:1337. doi: 10.1186/s12889-019-7713-1 (PMC6805482; doi:10.1186/s12889-019-7713-1)
Supplement: Supplementary file 2 — Additional file 2: Table S2. Database terms of search. [file 12889_2019_7713_MOESM2_ESM.docx]

**Table S2**. Database terms of search

| **PubMed (N=722)** |
| --- |
| ("Burnout, Psychological"[Mesh] OR "Burnout, Professional"[Mesh] OR "Compassion Fatigue"[Mesh] OR burnout[tiab] OR burning out[tiab] OR burn out[tiab] OR emotional exhaustion[tiab] OR secondary trauma*[tiab] OR vicarious trauma*[tiab] OR compassion fatigue[tiab])  AND  ("Middle East"[Mesh] OR lebanon[all fields] OR syria[all fields] OR jordan[all fields] OR iraq[all fields] OR turkey[all fields] OR iran[all fields] OR saudi[all fields] OR oman[all fields] OR yemen[all fields] OR egypt[all fields] OR israel[all fields] OR palestine[all fields] OR united arab emirates[all fields] OR qatar[all fields] OR bahrain[all fields] OR kuwait[all fields]) |
| **PsycINFO (N=1167)** |
| (DE (“Occupational Stress” OR “Compassion Fatigue”) OR TI (“burnout” OR “burning out” OR “burn out” OR “emotional exhaustion” OR “secondary trauma*” OR “vicarious trauma*” OR “compassion fatigue”) OR AB (“burnout” OR “burning out” OR “burn out” OR “emotional exhaustion” OR “secondary trauma*” OR “vicarious trauma*” OR “compassion fatigue”))  AND  (TX (“middle east” OR “lebanon” OR “syria” OR “jordan” OR “iraq” OR “turkey” OR “iran” OR “saudi” OR “oman” OR “yemen” or “egypt” OR “israel” OR “palestine” OR “united arab emirates” OR “qatar” OR “bahrain” OR “kuwait”)) |
| **Web of Science (N=645)** |
| TS=("burnout" OR "burning out" OR "burn out" OR "emotional exhaustion" OR "secondary trauma*" OR "vicarious trauma*" OR "compassion fatigue")  AND  TS=("middle east” OR “lebanon” OR “syria” OR “jordan” OR “iraq” OR “turkey” OR “iran” OR “saudi” OR “oman” OR “yemen” OR “egypt” OR “israel” OR “palestine” OR “united arab emirates” OR “qatar” OR “bahrain” OR “kuwait”) |
