# Supplementary material for: Burnout among healthcare providers in the complex environment of the Middle East: a systematic review
Source: BMC Public Health. 2019 Oct 22;19:1337. doi: 10.1186/s12889-019-7713-1 (PMC6805482; doi:10.1186/s12889-019-7713-1)
Supplement: Supplementary file 3 — Additional file 3: Table S3. Quality assessment based on modified Newcastle-Ottawa Scale of studies on burnout among physicians in the Middle East (N = 54). [file 12889_2019_7713_MOESM3_ESM.docx]

**Supplementary Table 3.** Quality assessment based on modified Newcastle-Ottawa Scale of studies on burnout among physicians in the Middle East (N = 54).

| **Study Characteristics** | | **Newcastle-Ottawa Scale†** | | | | | | | | |
| --- | --- | --- | --- | --- | --- | --- | --- | --- | --- | --- |
| **First author and year** | **Type of study** | **Selection** | | | **Comparability** | | | **Exposure/Outcome** | | |
| Abdulrahman, 2018 | cross-sectional | *** | | | - | | | ** | | |
| Abut, 2012 | cross-sectional | *** | | | - | | | ** | | |
| Afana, 2017 | cross-sectional | ** | | | - | | | ** | | |
| Agha, 2015 | cross sectional | ** | | | * | | | ** | | |
| Ahmadpanah, 2015 | cross sectional | **** | | | - | | | ** | | |
| Aksoy, 2014 | cross-sectional | ** | | | - | | | * | | |
| Al-Dubai, 2010 | cross sectional | ** | | | - | | | * | | |
| Al-Mendalawi, 2018 | cross-sectional | ** | | | - | | | * | | |
| Al-Sareai, 2013 | cross sectional | ** | | | - | | | ** | | |
| Al-Shuhail, 2017 | cross sectional | * | | | - | | | * | | |
| Al-Youbi, 2013 | cross sectional | ** | | | - | | | * | | |
| Aldrees, 2015 | cross sectional | ** | | | - | | | ** | | |
| Aldrees, 2017 | cross sectional | ** | | | - | | | ** | | |
| Aldrees, 2013 | cross sectional | *** | | | ** | | | ** | | |
| Alyamani, 2018 | cross sectional | ** | | | - | | | * | | |
| Amiri, 2016 | cross sectional | ** | | | * | | | ** | | |
| Arvandi, 2016 | cross-sectional | *** | | | * | | | ** | | |
| Ashkar, 2010 | cross sectional | ** | | | * | | | ** | | |
| Bawakid, 2017 | cross sectional | *** | | | ** | | | ** | | |
| Ben-Itzhak, 2015 | cross sectional | ** | | | - | | | ** | | |
| Capraz, 2017 | cross sectional | * | | | * | | | ** | | |
| Erdur, 2015 | cross sectional | *** | | | ** | | | ** | | |
| Granek, 2016 | cross-sectional | * | | | * | | | ** | | |
| Granek, 2017 | cross-sectional | * | | | ** | | | ** | | |
| Grossman, 2019 | cross-sectional | ** | | | - | | | ** | | |
| Gül, 2017 | cross-sectional | * | | | - | | | * | | |
| Haber, 2013 | cross-sectional | ** | | | - | | | ** | | |
| Haik, 2017 | cross sectional | ** | | | - | | | ** | | |
| Hameed, 2018 | cross-sectional | * | | | - | | | ** | | |
| Hasan, 2015 | cross sectional | ** | | | - | | | * | | |
| Jalili, 2013 | cross sectional | ** | | | - | | | ** | | |
| Jamjoom, 2018 | cross sectional | *** | | | - | | | ** | | |
| Karaoglu, 2015 | cross sectional | *** | | | - | | | ** | | |
| Keinan, 1987 | cross sectional | ** | | | - | | | ** | | |
| Kosan, 2018 | cross sectional | **** | | | * | | | ** | | |
| Kotb, 2014 | cross sectional | ***** | | | * | | | ** | | |
| Kushnir, 2006 | cross sectional | ** | | | - | | | ** | | |
| Kushnir, 2008 | cross sectional | ** | | | - | | | ** | | |
| Kushnir, 2014 | cross sectional | * | | | - | | | ** | | |
| Pirincci, 2015 | cross sectional | ** | | | - | | | ** | | |
| Sadat-Ali, 2005 | cross sectional | ** | | | - | | | ** | | |
| Salem, 2018 | cross sectional | ** | | | * | | | ** | | |
| Salem, 2018 | cross sectional | ** | | | - | | | ** | | |
| Shams, 2013 | cross sectional | *** | | | * | | | ** | | |
| Shinan-Altman, 2018 | cross sectional | *** | | | * | | | ** | | |
| Soltanifar, 2018 | cross sectional | ** | | | - | | | ** | | |
| Talih, 2016 | cross sectional | ** | | | * | | | ** | | |
| Tarcan, 2017 | cross sectional | ** | | | * | | | ** | | |
| Taycan, 2014 | cross sectional | ** | | | - | | | ** | | |
| Turgut, 2016 | cross-sectional | ** | | | - | | | ** | | |
| Tzischinsky, 2011 | cross-sectional | ** | | | - | | | ** | | |
|  |  | **Joanna Briggs Critical Appraisal Checklist** | | | | | | | | |
|  |  | **Q1** | **Q2** | **Q3** | **Q4** | **Q5** | **Q6** | **Q7** | **Q8** | **Q9** |
| Bar-Sela, 2012 | Quasi-experimental | Y | Y | NA | N | N | NA | Y | Y | Y |
| Ghannam, 2019 | Quasi-experimental | Y | Y | NA | N | N | NA | Y | Y | Y |
| Kotb, 2014 | Quasi-experimental | Y | NA | NA | N | N | NA | NA | Y | Y |

**†** Study quality was assessed using a modified NOS for cross-sectional studies (Herzog et al., 2013) and Joanna Briggs Critical Appraisal Checklist for quasi-experimental studies (Tufanaru 2017).

Cross-sectional study maximum score: Selection (5), Comparability (2), Outcome (3); Total = 10
