# Supplementary material for: Burnout among healthcare providers in the complex environment of the Middle East: a systematic review
Source: BMC Public Health. 2019 Oct 22;19:1337. doi: 10.1186/s12889-019-7713-1 (PMC6805482; doi:10.1186/s12889-019-7713-1)
Supplement: Supplementary file 4 — Additional file 4: Table S4. Quality assessment based on modified Newcastle-Ottawa Scale of studies on burnout among nurses in the Middle East (N = 53). [file 12889_2019_7713_MOESM4_ESM.docx]

**Table S4.** Quality assessment based on modified Newcastle-Ottawa Scale of studies on burnout among nurses in the Middle East (N = 53).

| **Study Characteristics** | | **Newcastle-Ottawa Scale†** | | | | | | | | |
| --- | --- | --- | --- | --- | --- | --- | --- | --- | --- | --- |
| **First author and year** | **Type of study** | **Selection** | | | **Comparability** | | | **Exposure/Outcome** | | |
| Abushaikha, 2009 | cross sectional | *** | | | * | | | ** | | |
| Ahmadi, 2014 | cross sectional | *** | | | - | | | ** | | |
| Akkus, 2010 | cross-sectional | ** | | | - | | | ** | | |
| Akman, 2016 | cross-sectional | *** | | | * | | | ** | | |
| Al-Turki, 2010 | cross-sectional | ** | | | - | | | ** | | |
| Al-Turki, 2010 | cross sectional | ** | | | - | | | ** | | |
| Alharbi, 2016 | cross sectional | *** | | | * | | | ** | | |
| Alimoglu, 2005 | cross sectional | ** | | | - | | | ** | | |
| Altun, 2002 | cross sectional | ** | | | - | | | ** | | |
| Anwar, 2017 | cross sectional | *** | | | * | | | ** | | |
| Arslan, 2016 | cross sectional | ** | | | - | | | ** | | |
| Azmoon, 2018 | cross sectional | ** | | | - | | | ** | | |
| Bagheri, 2019 | cross sectional | **** | | | * | | | ** | | |
| Bakir, 2010 | cross sectional | ** | | | - | | | ** | | |
| Chayu, 2011 | cross sectional | *** | | | * | | | ** | | |
| Darban, 2016 | cross sectional | ** | | | * | | | ** | | |
| Demir, 2003 | cross sectional | **** | | | * | | | ** | | |
| Dor, 2018 | cross sectional | ** | | | - | | | ** | | |
| Emold, 2011 | cross sectional | ** | | | - | | | ** | | |
| Farahbod, 2015 | cross sectional | *** | | | * | | | ** | | |
| Gholami, 2016 | cross sectional | *** | | | * | | | ** | | |
| Hamaideh, 2011 | cross sectional | ** | | | * | | | * | | |
| Iecovich, 2017 | cross sectional | *** | | | * | | | ** | | |
| Ilhan, 2008 | cross sectional | ** | | | - | | | ** | | |
| Kapucu, 2009 | cross sectional | ** | | | - | | | ** | | |
| Karadag, 2017 | cross sectional | ** | | | - | | | ** | | |
| Karakoc, 2016 | cross sectional | *** | | | * | | | ** | | |
| Karaman, 2017 | cross sectional | ** | | | - | | | ** | | |
| Kavurmacı, 2014 | cross sectional | ** | | | - | | | ** | | |
| Kutluturkan, 2016 | cross sectional | ** | | | * | | | ** | | |
| Kızılcı, 2012 | cross sectional | * | | | - | | | * | | |
| Moghaddasi, 2013 | cross sectional | **** | | | - | | | ** | | |
| Mohammad, 2012 | cross sectional | *** | | | - | | | ** | | |
| Mudallal, 2017 | cross sectional | **** | | | * | | | ** | | |
| Nayeri, 2009 | cross sectional | **** | | | - | | | ** | | |
| Ozden, 2013 | cross sectional | ** | | | - | | | ** | | |
| Özgür, 2018 | cross sectional | ** | | | - | | | ** | | |
| Palazoglu, 2017 | cross sectional | ** | | | - | | | ** | | |
| Rezaei, 2018 | cross sectional | **** | | | - | | | ** | | |
| Ron, 2014 | cross sectional | * | | | - | | | ** | | |
| Sahraian, 2008 | cross sectional | ** | | | - | | | ** | | |
| Shahriari, 2014 | cohort | *** | | | * | | | * | | |
| Shamali, 2015 | cross sectional | **** | | | - | | | ** | | |
| Sorour, 2012 | cross sectional | ** | | | - | | | ** | | |
| Soroush, 2016 | cross sectional | ** | | | - | | | ** | | |
| Taleghani, 2017 | cross sectional | ** | | | - | | | ** | | |
| Tekindal, 2012 | cross sectional | ** | | | - | | | ** | | |
| Topbas, 2019 | cross sectional | ** | | | - | | | ** | | |
| Tuna, 2014 | cross sectional | *** | | | - | | | ** | | |
| Yousefy, 2006 | cross sectional | **** | | | - | | | ** | | |
|  |  | **Cochrane Risk of Bias Tool†** | | | | | | | | |
| Gunusen, 2010 | RCT | fair quality | | | | | | | | |
| Ozbas, 2016 | RCT | fair quality | | | | | | | | |
|  |  | **Joanna Briggs Critical Appraisal Checklist** | | | | | | | | |
|  |  | **Q1** | **Q2** | **Q3** | **Q4** | **Q5** | **Q6** | **Q7** | **Q8** | **Q9** |
| Sabanciogulari, 2015 | quasi-experimental | Y | Y | Y | Y | N | Y | Y | Y | Y |

**†** Study quality was assessed using a modified NOS for cross-sectional studies (Herzog et al., 2013) and a NOS scale for cohort studies (Wells 1999) and the Cochrane Risk of Bias Tool for randomized controlled trials (Higgins et al., 2011) and Joanna Briggs Institute Critical Appraisal Checklist for quasi-experimental studies (Tufanaru 2017).

Cross-sectional study maximum score: Selection (5), Comparability (2), Outcome (3); Total = 10

Cohort study maximum score: Selection (4), Comparability (2), Outcome (3); Total = 9
