# Supplementary material for: Burnout among healthcare providers in the complex environment of the Middle East: a systematic review
Source: BMC Public Health. 2019 Oct 22;19:1337. doi: 10.1186/s12889-019-7713-1 (PMC6805482; doi:10.1186/s12889-019-7713-1)
Supplement: Supplementary file 5 — Additional file 5: Table S5. Quality assessment based on the Newcastle-Ottawa Scale on burnout among healthcare workers in the Middle East (N = 22). [file 12889_2019_7713_MOESM5_ESM.docx]

**Table S5.** Quality assessment based on the Newcastle-Ottawa Scale on burnout among healthcare workers in the Middle East

(N = 22).

| **Study Characteristics** | | **Newcastle-Ottawa Scale†** | | |
| --- | --- | --- | --- | --- |
| **First author and year** | **Type of study** | **Selection** | **Comparability** | **Exposure/Outcome** |
| Abdo, 2016 | cross-sectional | *** | - | ** |
| Abarghouei, 2016 | cross sectional | ** | - | ** |
| Alacacioglu, 2009 | cross sectional | ** | - | ** |
| Alameddine, 2011 | cross sectional | *** | - | ** |
| Alameddine, 2012 | cross sectional | **** | * | ** |
| Alameddine, 2017 | cross sectional | **** | ** | ** |
| Ashtari, 2009 | cross sectional | *** | - | ** |
| Bijari, 2016 | cross sectional | *** | - | ** |
| Calgan, 2011 | cross sectional | **** | * | ** |
| Demirci, 2010 | cross sectional | ** | * | ** |
| Devebakan, 2018 | cross sectional | ** | * | ** |
| Gulalp, 2008 | cross-sectional | ** | - | ** |
| GÖKÇEN, 2013 | cross-sectional | ** | - | * |
| Guveli, 2015 | cross sectional | *** | ** | ** |
| Hamdan, 2017 | cross sectional | *** | * | ** |
| Hosseiniarzfuni, 2015 | cross sectional | ** | - | ** |
| Kabir, 2016 | cross sectional | *** | - | ** |
| Kömür, 2017 | cross sectional | ** | - | ** |
| Malakouti, 2011 | cross sectional | ** | - | * |
| Tarcan, 2017 | cross sectional | *** | ** | ** |
| Tekin, 2017 | cross sectional | ** | * | ** |
| Tunc, 2009 | cross-sectional | ** | * | ** |

**†** Study quality was assessed using a modified NOS for cross-sectional studies (Herzog et al., 2013)

Cross-sectional study maximum score: Selection (5), Comparability (2), Outcome (3); Total = 10
