# Supplementary material for: Burnout among healthcare providers in the complex environment of the Middle East: a systematic review
Source: BMC Public Health. 2019 Oct 22;19:1337. doi: 10.1186/s12889-019-7713-1 (PMC6805482; doi:10.1186/s12889-019-7713-1)
Supplement: Supplementary file 6 — Additional file 6: Table S6. Quality assessment based on the Newcastle-Ottawa Scale on burnout among midwives, medical and nursing students in the Middle East(N = 7). [file 12889_2019_7713_MOESM6_ESM.docx]

**Table S6.** Quality assessment based on the Newcastle-Ottawa Scale on burnout among midwives, medical and nursing students in the Middle East(N = 7).

| **Study Characteristics** | | **Newcastle-Ottawa Scale†** | | |
| --- | --- | --- | --- | --- |
| **First author and year** | **Type of study** | **Selection** | **Comparability** | **Exposure/Outcome** |
| Al-Alawi, 2017 | cross sectional | **** | ** | ** |
| Almalki, 2017 | cross sectional | *** | - | ** |
| Altannir, 2019 | cross sectional | *** | - | ** |
| Ebrahimi, 2018 | cross sectional | ** | - | ** |
| Fares, 2016 | cross sectional | *** | * | ** |
| Sevencan, 2010 | cross sectional | *** | - | ** |
| Talih, 2018 | cross sectional | *** | * | ** |

**†** Study quality was assessed using a modified NOS for cross-sectional studies (Herzog et al., 2013)

Cross-sectional study maximum score: Selection (5), Comparability (2), Outcome (3); Total = 10
